# QTL mapping for aluminum tolerance in RIL population of soybean (*Glycine max* L.) by RAD sequencing

**DOI:** 10.1371/journal.pone.0223674

**Published:** 2019-10-29

**Authors:** Xinxin Wang, Yanbo Cheng, Ce Yang, Cunyi Yang, Yinghui Mu, Qiuju Xia, Qibin Ma

**Affiliations:** 1 The State Key Laboratory for Conservation and Utilization of Subtropical Agro-bioresources, South China Agricultural University, Guangzhou, Guangdong, China; 2 The Key Laboratory of Plant Molecular Breeding of Guangdong Province, College of Agriculture, South China Agricultural University, Guangzhou, Guangdong, China; 3 The National Engineering Research Center of Plant Space Breeding, South China Agricultural University, Guangzhou, Guangdong, China; 4 The Beijing Genomics Institute (BGI)-Shenzhen, Shenzhen, China; New South Wales Department of Primary Industries, AUSTRALIA

## Abstract

Aluminum (Al^3+^) toxicity is a typical abiotic stress that severely limits crop production in acidic soils. In this study, an RIL (recombinant inbred line, F_12_) population derived from the cross of Zhonghuang 24 (ZH 24) and Huaxia 3 (HX 3) (160 lines) was tested using hydroponic cultivation. Relative root elongation (RRE) and apical Al^3+^ content (AAC) were evaluated for each line, and a significant negative correlation was detected between the two indicators. Based on a high-density genetic linkage map, the phenotypic data were used to identify quantitative trait loci (QTLs) associated with these traits. With composite interval mapping (CIM) of the linkage map, five QTLs that explained 39.65% of RRE and AAC variation were detected on chromosomes (Chrs) Gm04, Gm16, Gm17 and Gm19. Two new QTLs, *qRRE_04* and *qAAC_04*, were located on the same region of bin93-bin94 on Chr Gm04, which explained 7.09% and 8.98% phenotypic variation, respectively. Furthermore, the results of the expression analysis of candidate genes in the five genetic regions of the QTLs showed that six genes (*Glyma*.*04g218700*, *Glyma*.*04g212800*, *Glyma*.*04g213300*, *Glyma*.*04g217400*, *Glyma*.*04g216100* and *Glyma*.*04g220600*) exhibited significant differential expression between the Al^3+^ treatment and the control of two parents. The results of qRT-PCR analysis indicated that *Glyma*.*04g218700* was upregulated by Al^3+^ treatment with the hundreds-fold increased expression level and may be a candidate gene with potential roles in the response to aluminum stress. Therefore, our efforts will enable future functional analysis of candidate genes and will contribute to the strategies for improvement of aluminum tolerance in soybean.

## Introduction

Aluminum (Al^3+^) toxicity is one of the major factors affecting crop production on acidic soils worldwide [1, 2]. When the soil pH decreases to values less than 5.0, Al is solubilized as the phytotoxic Al^3+^, which has a pernicious effect on crops. It was found that root elongation can be inhibited in seconds at micromolar concentrations of Al^3+^ [3]. The primary location of Al^3+^ toxicity is at the root tip where Al^3+^ binds to the cell wall [4]. Changes in some components of the cell wall lead to a limited capacity of damaged roots for absorption of sufficient water and nutrients from soil [2]. Additionally, the damaged root impeded the growth of shoot, and eventually reduced the yield of crops. Soybean is one of the most important crops in the subtropical zone and is also damaged by Al^3+^ toxicity in acidic soil. Hence, investigation of the traits associated with Al^3+^ toxicity via a combination of identified soybean germplasms and sequencing technology is of great significance.

It is well known that two types of mechanisms of Al^3+^ resistance in soybean are involved in the exclusion of Al^3+^ from the root apex (external exclusion) or in conferring tolerance to Al^3+^ in the plant symplast (internal tolerance) [5]. The mechanism of external exclusion involves excretion of organic acids to chelate Al^3+^ from the root cells, increasing the rhizospheric pH and external exclusion of border cells [6, 7]. However, the mechanism of internal tolerance depends on chelation of organic acids and segregation of Al^3+^ in vacuoles. Antioxidant metabolism as well as hormone signal transduction also contribute to aluminum tolerance [8, 9].

Aluminum tolerance of soybean is a complex quantitative trait with substantial genetic variation [10]. Studies on the genetic architecture of soybean aluminum tolerance remain challenging due to the interactions of environments and genotypes. Conventional breeding has relied on the selection of highly Al^3+^-tolerant cultivars for crop improvement, but this method is costly and time consuming [11]. In recent years, genome-wide association study (GWAS) and QTL mapping are commonly used to map genetic markers associated with quantitative traits. GWAS analysis generally involves the natural populations to detect the correlation between genetic polymorphism and phenotypic variation by statistical methods based on the linkage disequilibrium. A number of important GWAS loci and candidate genes for Al^3+^-tolerant traits have been reported over the recent decade [12]. Meanwhile, the strategy of QTL mapping has provided an improved understanding of the genetic architectures of complex traits, which has accelerated crop improvement [13]. Accordingly, extensive efforts have been directed at QTL mapping for aluminum tolerance in *Arabidopsis thaliana* [14] and several crops, including rice [15, 16], wheat [17, 18], barley [19], maize [20], soybean [21] and alfalfa [22].

In soybean, some QTLs of aluminum tolerance have been identified using populations from different genetic backgrounds, for which the traits of root elongation were often used to represent aluminum tolerance. In the early 2000s, a genetic linkage map containing 155 restricted fragment length polymorphism (RFLP) markers was constructed using the population derived from Young × PI 416937. Bianchihall *et al*. [23] detected the genetic basis of Al-tolerant traits in soybean using the map and indicated five independent RFLP markers associated with root elongation. Qi et al. [20] and Korir *et al*. [24] focused on the progenies of Kefeng No.1 × Nannong 1138–2 and used genetic linkage map with RFLP and simple sequence repeats (SSR) markers to detect one major and two minor QTLs for aluminum tolerance. In general, the explorations of QTL mapping indicated that approximately two to five dominant loci controlled the variation in Al-tolerance levels [25, 26].

However, the traditional molecular markers, including RFLP, SSR and amplified fragment length polymorphism (AFLP), exhibited low density and uneven distribution throughout the genome [27]. QTL mapping of complex quantitative traits such as aluminum tolerance on soybean remains elusive due to the limited efficiency and accuracy of QTL positioning. In recent years, single-nucleotide polymorphism (SNP) markers have emerged with the assistance of high-throughput sequencing technology and have been mapped across plant genomes with high density and relatively even distributions, thereby improving the accuracy of QTL mapping. Over the last few years, high-density genetic maps have been constructed using recombination bins as markers [28]. Restriction-site-associated DNA sequencing (RAD-seq) [29], one of the next-generation sequencing (NGS) methods [30], has been effectively used for high-density SNP marker discovery and QTL analysis [31, 32]. In barley and wheat, high-density genetic maps have been established using RAD-seq technology with hundreds of thousands of SNP markers as well as other polymorphic markers [33]. Abdel-Haleem *et al*. improved the linkage map using the progenies derived from the cross of Young and PI416937 and further developed *Glyma08g42400*-SNP as a major QTL to be used for marker-assisted selection of aluminum tolerance [34]. Recently, a high-density genetic linkage map based on RAD-seq technology was constructed to map QTLs for both yield-related and quality traits [35, 36]. The genetic maps with ultrahigh density for the complex polyploid crops with DNA markers indicate that RAD-seq technology can be practically applied to identify the genetic basis of complex quantitative traits.

The objectives of the present study were to develop a high-density genetic map using bin markers with RAD-seq technology to identify QTLs for the traits of aluminum tolerance in the F_12_ RIL population derived from the cross of Zhonghuang 24 (ZH 24) and Huaxia 3 (HX 3) and to analyse candidate genes that may influence aluminum tolerance using Gene Ontology (GO) enrichment analysis.

## Materials and methods

### Plant materials

An RIL population with 160 lines of the F_12_ generation derived from a cross between ZH 24 (female parent) and HX 3 (male parent) was used in the current study. ZH 24 is an Al^3+^-sensitive cultivar derived from Fendou 31 × Zhongdou 19, while HX 3 is an Al^3+^-tolerant cultivar derived from Guizao 1 × BRSMG68 (a high-yield Brazilian cultivar) [35]. All the F_12_ lines of the RIL population and their parents were provided by the Guangdong Subcenter of National Center for Soybean Improvement, South China Agricultural University.

### Experiment trial design for phenotyping

A preliminary test was designed to determine the appropriate concentration of Al^3+^ and Al^3+^ treatment for hydroponic cultivation. The two parents and five randomly selected lines (L10, L70, L154, L206, and L245) were used to identify Al^3+^ tolerance with RRE as a detection index. The concentrations of AlCl_3_ (0.5 mM CaCl_2_, pH 4.5) were set as 0, 5, 15, 20, 25 and 30 μΜ. The RRE of each line and parents was measured by imaging analysis during successive treatment periods of 24 h, 48 h and 72 h. The Al^3+^ concentration and duration that provided the widest separation among these lines were chosen for screening the RIL population.

The phenotype of the RIL population was estimated by the RRE and AAC after hydroponic cultivation along with the parents. For each line as well as the two parents, the hydroponic experiments were carried out with three replications. For each replication, 6 seedlings with nearly the same root length (approximately 8 cm) were fixed using sponge in the holes of foam floating plate in plastic containers either with or without AlCl_3_ treatment (0.5 mM CaCl_2_, pH 4.5). The average values of phenotypic data for RRE and AAC were used for mapping and identifying QTLs for aluminum tolerance.

### Hydroponics and trait measurement

A total of 80–100 plump seeds of each line and the parents were germinated in sterilized vermiculite for three days at 26°C in continuous darkness. Six seedlings with nearly the same root length were then held in foam support floats that were suspended in 2.5-L plastic containers without Al^3+^ for acclimation to hydroponic conditions (0.5 mM CaCl_2_, pH 4.5, 16 h light/8 h dark). After 24 h of acclimation, the seedlings were photographed carefully using a camera (Nikon, COOLPIX A1000) to determine the main root lengths with a ruler beside them as scale. Then, the seedlings were transferred to solutions with or without AlCl_3_ (0.5 mM CaCl_2_, pH 4.5). The roots of the seedlings were photographed again after Al^3+^ exposure. To ensure the accuracy of the two kinds of measurements before and after Al^3+^ exposure, we marked the root at the initial position of the measurement. During the process of cultivation, the nutrient solution was aerated constantly with a flexible pipe connected with air pump.

The main root lengths were determined from the photographs using ImageJ software (National Institutes of Health, http://imagej.nih.gov/ij/). Root elongation was defined as the difference between the initial length before Al^3+^ treatment and the final length after Al^3+^ treatment. The root elongation under control (REC) and the root elongation under Al^3+^- stress (REA) were calculated, and the RRE was equal to REA/REC ×100% [37].

After Al^3+^ treatment, apical roots (0–2 cm) were excised by a scalpel, washed three times with 0.5 mM CaCl_2_ solution, and dried on filter paper. Then, six root tips for each line and the two parents were placed in a microcentrifuge tube (1.5 ml) containing 1.0 ml of 2 M HCl and extracted for 48 h with continuous shaking to release Al^3+^ from the soybean roots. The Al^3+^ levels in the extracts were determined by inductively coupled plasma-optical emission spectrometry (ICP-OES) (VARIAN 710-ES, America) [38].

### Genetic map and QTL detection

#### SNP genotyping

Genotyping was carried out as previously described [35]. The soybean reference genome from Williams 82 [39] was used for read mapping be comparison with the tag sequence by SOAP software (The Beijing Genomics Institute, http://soap.genomics.org.cn/). Input data for SNP calling with realSFS was prepared by SAMtools [40]. RealSFS was used for SNP calling of every locus in the RIL population. The likelihoods of genotypes for each individual were integrated and extracted as candidate SNPs that were then filtered using the following criteria: 40 ≤ depth ≤ 2500, sites with a probability ≥ 95%. These highly reliable SNPs were used to obtain the genotypes of the parents and the RIL population. Moreover, the genotypes of all SNPs from the soybean genome were analyzed by the sliding window method and further used for each individual to generate bin information. Finally, a fine genetic map including 3,426 bin markers was constructed using MSTMap (http://alumni.cs.ucr.edu/yonghui/mstmap.html) and MapChart software (Wageningen University, https://www.wur.nl/en/show/Mapchart.htm) [41].

#### QTL analysis

A high-density genetic map was constructed as previously described [35]. Composite interval mapping (CIM) was performed to detect QTLs using WinQTLCart software (North Carolina State University, http://statgen.ncsu.edu/qtlcart/WQTLCart.htm). The significant LOD threshold of 2.5 for QTLs was determined by a genome-wide permutation test with 1000 replications at the 5% level of significance. The analysis results also showed the effects of QTLs, the explanation rate of the phenotypic variation by QTLs and the interactions of QTLs. QTL mapping results were comprehensively compared to those published on Soybase (http://www.soybase.org/) [35].

#### Gene detection among the QTLs

The genes within all the QTL regions were listed by the Soybase website (http://www.soybase.org/). In addition, data from NCBI (https://www.ncbi.nlm.nih.gov/) and Phytozome (https://phytozome.jgi.doe.gov/pz/portal.html) were used to ascertain the conserved domains of the proteins and the possible functions of these domains. Specific primers for RT-PCR of these genes were designed using Primer Premier 5 software (PREMIER Biosoft, http://www.premierbiosoft.com/primerdesign/index.html).

### RNA extraction

The hydroponic conditions for cultivation of soybean seedlings were the same as those used for genotype analysis, as described in the section titles “Hydroponics and trait measurement”. Samples of the apical roots (0–2 cm) of the two parents were obtained and immediately frozen by using liquid nitrogen. Total RNA was extracted from the apical roots of seedlings grown under Al^3+^ stress or the control treatment using TRIzol reagent (TIANGEN, China). First-strand cDNA was synthesized using the PrimeScript™ RT Reagent Kit with gDNA Eraser (TAKARA, China) and used for further analysis of expression patterns for candidate genes.

### Gene expression assays

The RT-PCR assay was carried out to analyze the expression of the genes in the apical roots from the two parent seedlings, with the soybean *β-Tubulin* gene as an internal reference, with the specific primers 5′-AACCTCCTCCTCATCGTACT-3′ and 5′-GACAGCATCAGCCATGTTCA-3′ [42]. The total volume of the PCR mixture was 20 μl, containing 1 μl of first-strand cDNA, 1 μl of each primer, 7 μl of ddH_2_O, and 10 μl of the mixture containing Taq DNA polymerase. The amplification reaction was performed as follows: predenaturation at 95°C for 3 min, followed by 30 cycles (for almost all genes; for *β-Tubulin*, 26 cycles were used) of 15 s at 95°C, 15 s at 54°C and 30 s at 72°C min, with a final extension for 5 min at 72°C. The PCR products were separated by agarose gel electrophoresis. Furthermore, qRT-PCR was further used to analyze the expression of the candidate genes. All PCRs were performed in 20-μl reactions consisting of 1 μl of cDNA, 0.8 μM each gene-specific primer and a mixture from the SYBR Green Supermix Kit (Takara, Japan). The reaction conditions were as follows: predenaturation at 94°C for 3 min, followed by 40 cycles of denaturation at 94°C for 10 s and renaturation at 54°C for 10 s; at the end of the reaction, the system was maintained at 95°C for 10 s, followed by lowering the temperature to 65°C for 5 s. The soybean *Actin 3* gene [43] was used as an internal reference, with the forward primer 5′- GTGCACAATTGATGGACCAG-3′ and the reverse primer 5′-GCACCACCGGAGAGAAAATA-3′. Specific primers for RT-PCR and qRT-PCR of these genes were designed using Primer Premier 5 software (PREMIER Biosoft, http://www.premierbiosoft.com/primerdesign/index.html) (S1 and S2 Tables).

### Data analysis

Analysis of variance (ANOVAs) was performed using SAS 9.4 by the general linear model (GLM) procedure with a logarithmic transform of data if necessary [44]. The broad-sense heritability (*h*^*2*^) of RRE and AAC was calculated according to Knapp et al [45]. Heritability was calculated using the formula shown as follows: *h*^*2*^ = σ_g_^2^ / ((σ_e_^2^ / n) + σ_g_^2^), where σ_g_^2^ denotes the genetic variance; σ_e_^2^ denotes the error variance; and n denotes the replication number. The coefficient of variation was estimated as σ_g_/μ, where μ represents the mean value. Phenotypic Pearson’s correlations were calculated using the ‘PROC CORR’ option of the SAS program between the two different traits [45]. Linear regression analysis was plotted using ‘MASS’ and ‘car’ package of R 3.5.4.

## Results

### Phenotypic variation

**Fig 1:**
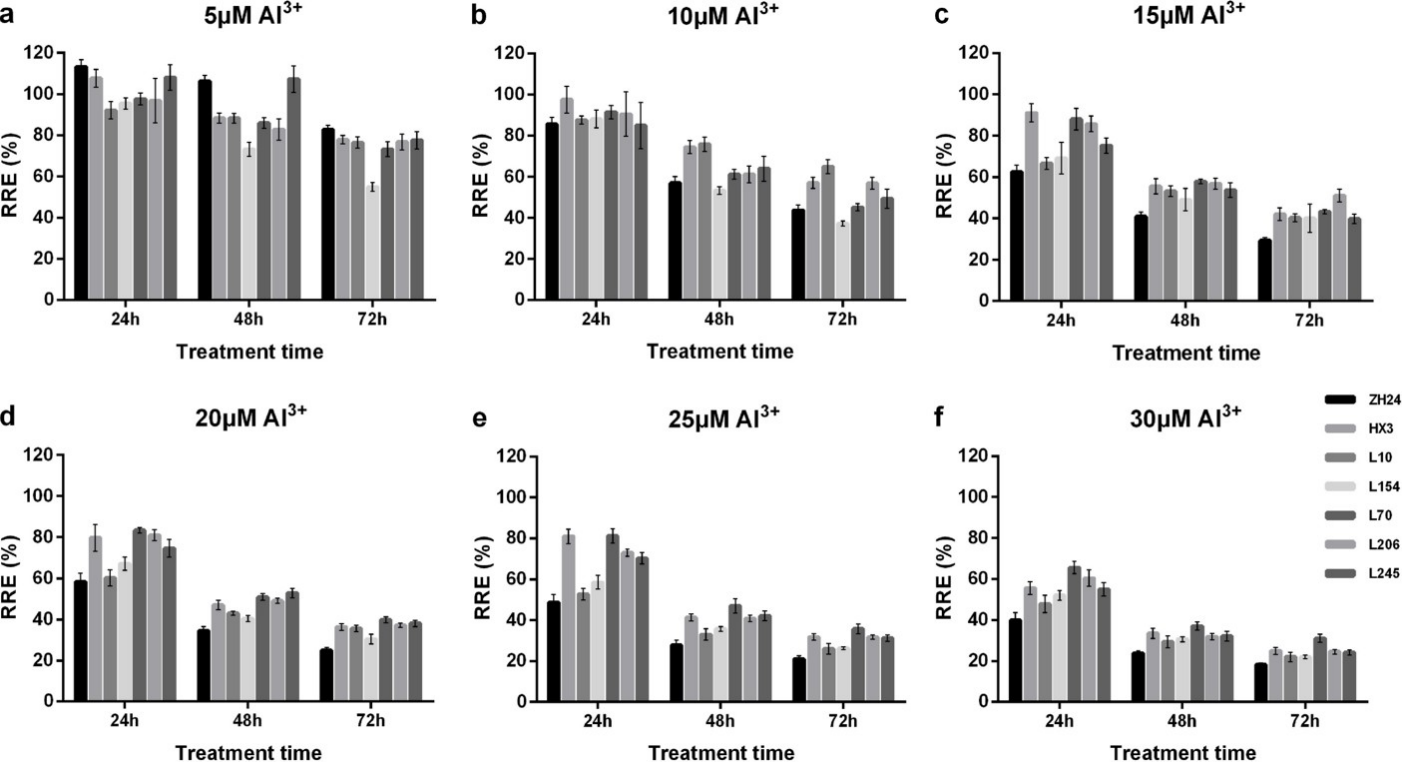
The responses of relative root elongation to Al^3+^ treatments of ZH24, HX3 and 5 RIL lines.

To explore the appropriate AlCl_3_ concentration and treatment time, two parents and five randomly selected lines were used to identify Al^3+^ tolerance characteristics. As shown in Fig 1, the RRE change trend was highly consistent among these lines. With increasing Al^3+^ concentrations, the RRE of each line decreased, showing strong inhibition of root elongation at high concentrations. Likewise, prolonged treatment of the lines led to decreased root elongation, so the 72-h treatment group exhibited the lowest RRE. In addition, the coefficient of variation (CV) was calculated to detect variations within each treatment. Comparative analysis showed that the condition with 25 μΜ [Al^3+^] and the 24-h treatment exhibited the highest CV (20.20%), which provided the greatest degree of dispersion among the five lines (Fig 1). Moreover, the two parents ZH 24 and HX 3 also showed the most significant difference under this condition (25 μΜ [Al^3+^], 24 h). Thus, 25 μΜ AlCl_3_ and 24 h of treatment were selected to obtain the widest separation in the RIL population.

**Fig 2:**
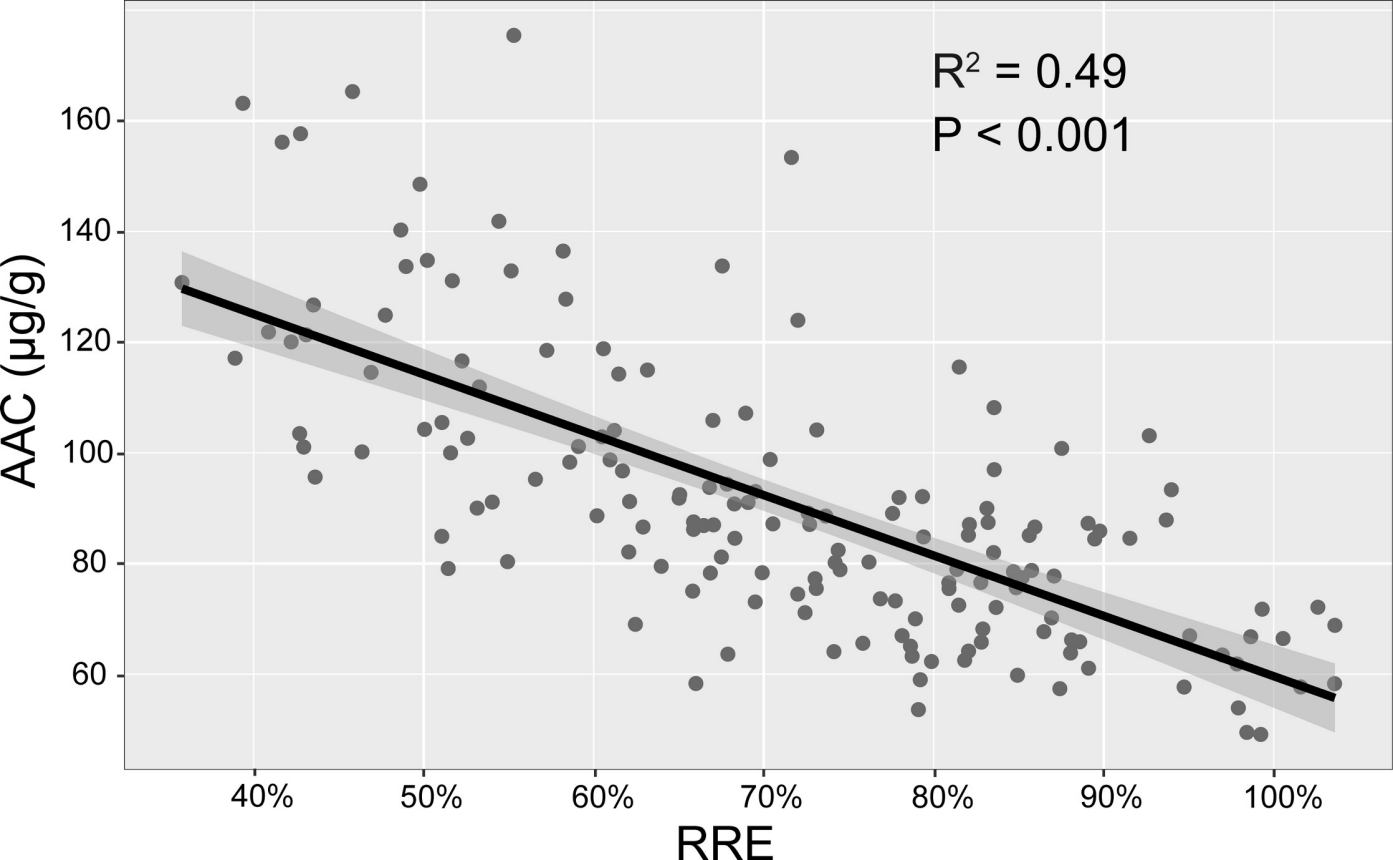
Relationship between RRE and AAC of RILs by linear regression analysis.

The ANOVA results demonstrated significant phenotypic differences among the RILs in RRE as well as AAC (P<0.01), but no significant differences among the three replications. The RRE results for all the lines showed a continuous distribution ranging from 34.78 to 103.60% among all the 160 F_12_ RILs with a mean of 71.26 ± 16.92% and CV of 23.74% (Tables 1 and S3). The average RREs of HX 3 and ZH 24 were 79.34% and 46.90%, respectively. Correspondingly, the AAC results showed a wide range from 49.11 to 175.46 μg/g in the RILs, with a high CV of 33.64%. The AAC value of ZH 24 was 114.55 μg/g while that of HX 3 was 92.10 μg/g (Tables 1 and S3). In addition, there was significantly negative correlation between RRE and AAC (spearman value -0.70) (Table 1). The linear regression analysis demonstrated that the AAC was significant negatively correlated with the RRE (R^2^ = 0.49, P < 0.001) (Fig 2).

**Table 1:** Phenotypic performance of Al^3+^ tolerance traits in two parents and RIL populations.

Traits^a^	Parents^b^		RILs^c^								
	ZH 24	HX 3	Mean	Min	Max	SD^d^	CV^e^	Heritability	Skewness	Kurtosis	r^f^
RRE (%)	46.90 ± 1.25	79.34 ± 0.23	71.37 ± 1.30	34.78	103.60	16.92	23.74	92.59%	-0.12	-0.78	-0.70**
AAC	114.55 ± 1.99	92.10 ± 4.33	90.83 ± 2.02	49.11	175.46	30.61	33.64	64.90%	0.98	0.77	

^a^ RRE: relative root elongation; AAC, apical Al^3+^ content.

^b^ Parents were cultivated and measured in each replicate of experiment, and the mean value is presented.

^c^ F_12_ RIL population size, n = 160, replicates r = 3, and the mean value is presented.

^d^ Standard deviation.

^e ^Coefficient of variation.

^f^ r, Correlation coefficient for phenotypic data between RRE and AAC

**P<0.01.

**Fig 3:**
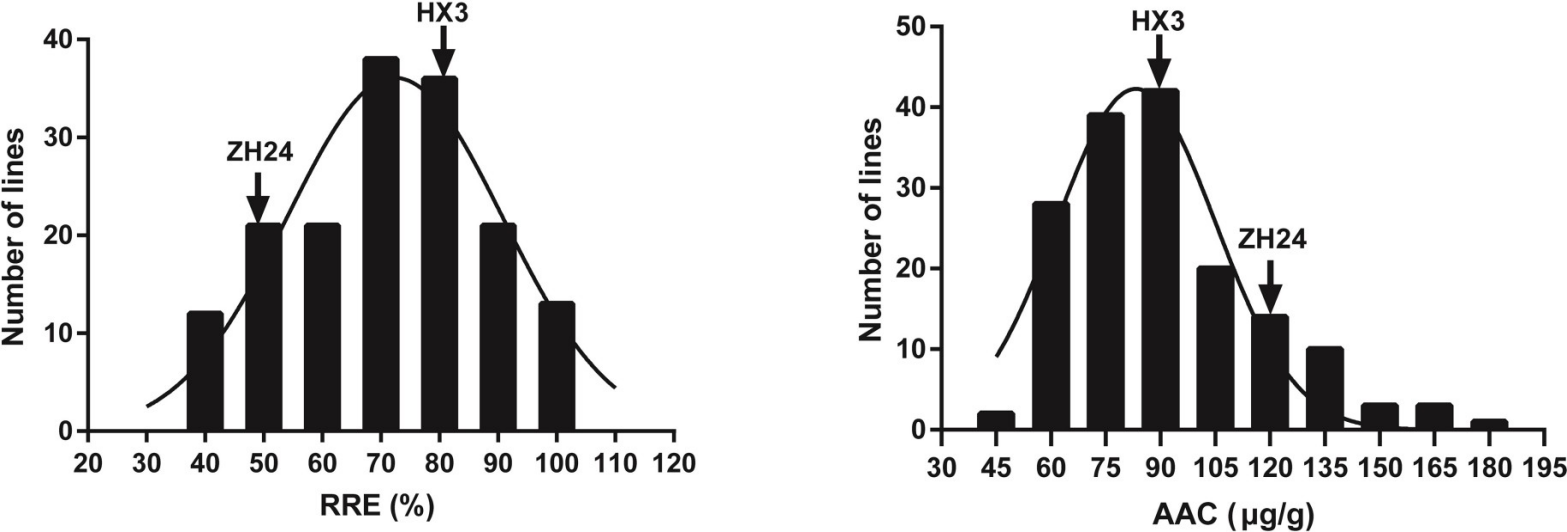
Frequency distribution of RRE and AAC among the RILs.

The frequency distribution of RRE and AAC is shown in Fig 3. The phenotypic data of the two traits (RRE and AAC) in the F_12_ lines under Al^3+^ stress (Table 1 and Fig 3) showed there were a normal distribution for RRE and a slightly skewed distribution for AAC with a large degree of separation. Moreover, the extensive transgressive segregation on either side of the parents in RRE and AAC indicated that the traits were inherited as quantitative characteristics and affected by multiple genetic factors (Table 1 and Fig 3). The estimated heritability of RRE and AAC were 92.59% and 64.90%, respectively (Table 1).

### Construction of the genetic linkage map

A total of 47,472 high-quality polymorphic SNPs were detected by genotypic analysis (S1 Fig). The recombination breakpoints for each individual were determined, and a total of 2,639 bin markers were obtained for the RILs (S2 Fig). The physical length of the bins ranged from 20.01 kb to 17.43 Mb with an average length of 360.01 kb. Using the 2,639 bins, a high-density linkage map was constructed, covering the genome length of 2638.24 cM with an average distance of 1.00 cM between adjacent markers (S1 File) [35]. Chi-square test of 2639 bin markers showed that 2356 markers (account for 89.28% of total markers) presented between parents with 1:1 segregation ratio (P>0.05), which was consistent with the characteristics of monogenic markers. There were 283 markers (account for 10.72%) showed separation distortion (P<0.05). In addition, most of the bin markers tend to be homozygous, and the heterozygous rate of bin markers was less than 4.79% (S4 Table). The linkage map was used for mapping analysis [35].

### QTL analysis

**Fig 4:**
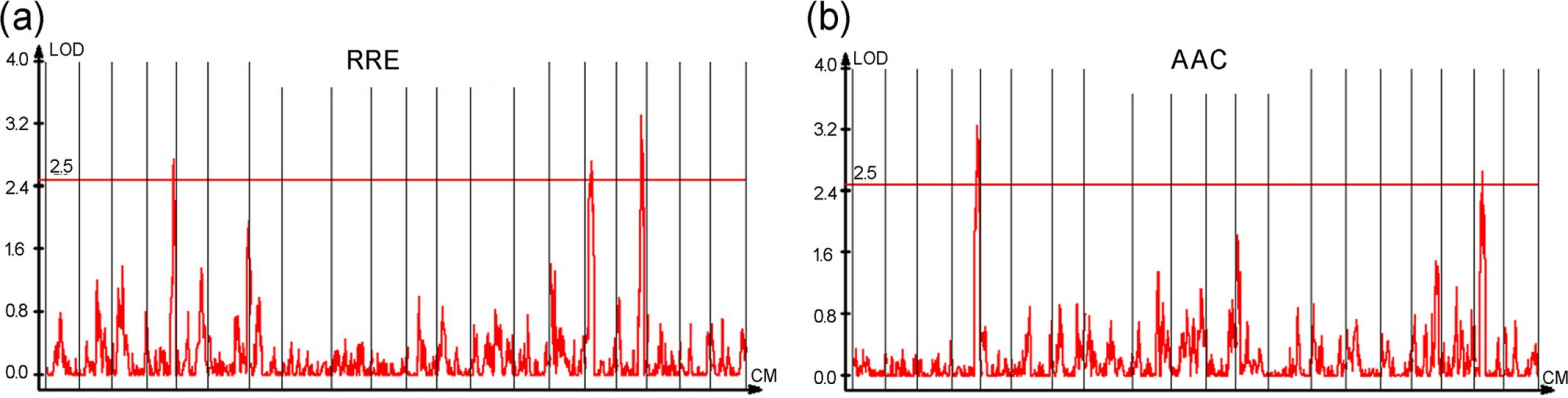
Distribution of LOD values of mapped QTLs of RRE (a) and AAC (b).

**Fig 5:**
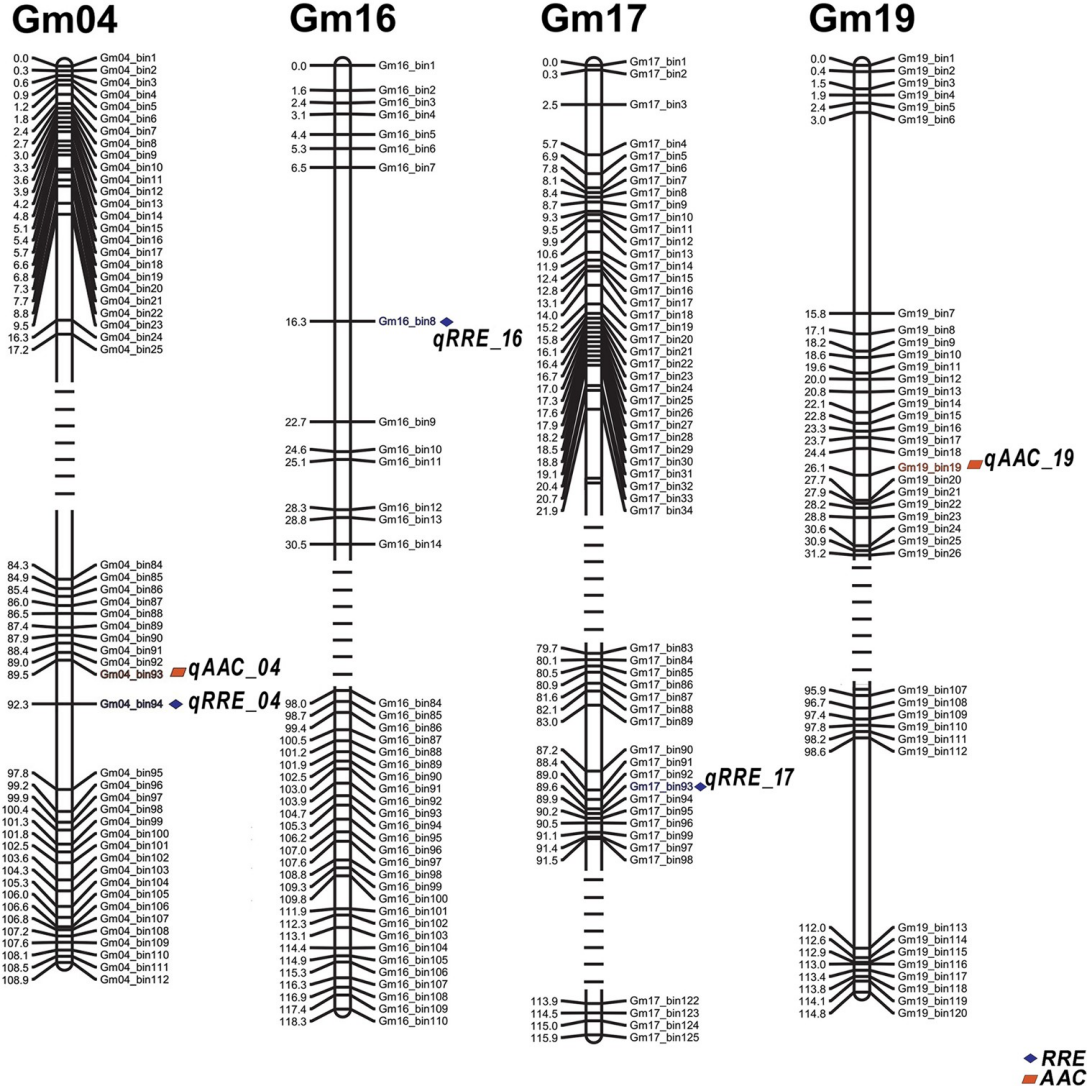
QTL positions on linkage groups of ZH 24 × HX 3. The virtual lines represent the truncated segments of chromosomes.

The results of CIM showed that five QTLs were detected on 4 chromosomes (Chr. Gm04, Gm16, Gm17, Gm19) (Table 2 and Figs 4 and 5). Three QTLs for RRE, namely, *qRRE_04*, *qRRE_16* and *qRRE_17*, were mapped on chromosomes Gm04, Gm16, and Gm17, with phenotypic variation (R^2^) explained by 7.09%-8.52% and LOD values ranging from 2.73 to 3.32 (Table 2). Two QTLs for AAC, namely, *qAAC_04* and *qAAC_19*, were identified, with phenotypic variation explained by 8.98% and 7.26% and LOD values of 3.27 and 2.66, respectively (Table 2). The comprehensive genetic effects explained by all QTLs for RRE and AAC were as high as 39.65%. Furthermore, the QTLs *qRRE_04* and *qAAC_04* were detected by the markers bin93-bin94 on Chr.04 in a genetic region between 90.50 and 92.30 cM (Table 2 and Fig 5), which indicated a new QTL on Chr.04 for the Al^3+^ tolerance trait of soybean root.

**Table 2:** QTLs for two traits identified by the CIM method in the RIL population.

Trait^a^	QTL name^b^	Chr.	Marker interval	Physical location (bp)	Physical distance (bp)	CI (cM)^c^	LOD^d^	Add^e^	R^2^(%)^f^
RRE	qRRE_04	Gm04	Gm04_bin93-bin94	45290936–46017212	726277	90.50–92.30	2.76	-0.05	7.09%
	qRRE_16	Gm16	Gm16_bin8	4038850–4099407	60558	20.30	2.73	-0.05	7.80%
	qRRE_17	Gm17	Gm17_bin93	38592282–38624563	32282	89.70	3.32	0.05	8.52%
AAC	qAAC_04	Gm04	Gm04_bin93-bin94	45290936–46017212	726277	90.50–92.30	3.27	7.72	8.98%
	qAAC_19	Gm19	Gm19_bin19	10253846–10556775	302930	27.10	2.66	6.85	7.26%

CIM: composite interval mapping.

^a^ RRE: relative root elongation; AAC: apical Al^3+^ content.

^b^ The QTL names are a composite of traits followed by the chromosome number.

^c^ Physical position corresponding to the 95% confidence interval for the detected QTL based on the Glyma.Wm82. a1. v1.1 gene model.

^d^ LOD indicates the logarithm of the odds score.

^e^ Additive effect of the alleles of parents.

^f^ R^2^ indicates the phenotypic variance explained by individual QTLs.

### Gene ontology (GO) enrichment analysis of genes in QTLs

The Soybase database (https://www.soybase.org/) was used to investigate effective candidate genes associated with aluminum tolerance. The analysis showed that 66 annotated genes were mapped on the regions of the five QTLs (S5 Table**)**. A total of 54 genes were detected in Gm04_bin93-bin94, from 45290936 bp to 46017212 bp. There were 6, 2 and 4 annotated genes in the three short intervals on Chr. Gm16 (Gm16_bin8), Gm17 (Gm17_bin93) and Gm19 (Gm19_bin19), respectively. To analyze the functional annotation of each gene, the AgriGO toolkit (http://bioinfo.cau.edu.cn/agriGO/index.php) was used to perform gene ontology (GO) analysis [46]. A total of 48 out of 66 genes were verified to have at least one GO annotation. All 48 genes were predicted to be involved in biological processes, cellular components or molecular functions. These genes could be grouped into seven categories, including cellular processes, biological regulation, metabolic processes, cell part, organelle, catalytic activity, and binding function (S6 Table**)**.

### Expression analysis of the candidate genes

**Fig 6:**
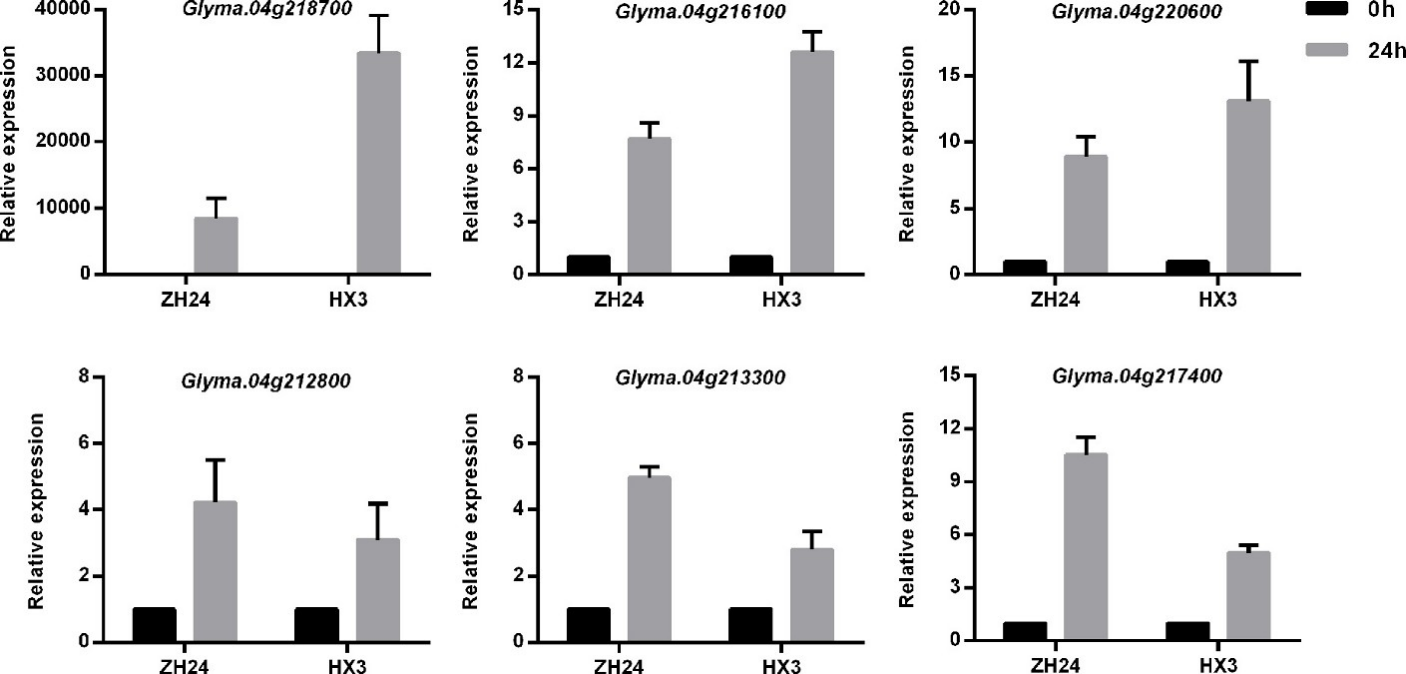
The relative expression of candidate genes by qRT-PCR in apical roots in both ZH 24 and HX 3 before (0 hrs) and after Al^3+^ exposure (24 hrs). The ordinate value represents the fold change in gene expression.

To investigate the responses of the annotated genes to aluminum stress, RT-PCR analysis was carried out using the two parents ZH 24 and HX 3 with or without Al^3+^ treatment. Fifteen differentially expressed genes were detected in the QTL regions of Chr. Gm04 and Gm16 (S3 Fig). Furthermore, qRT-PCR was used to analyze the expression patterns of these 15 annotated genes under Al^3+^ treatment. Most of the 15 annotated genes could respond to aluminum stress with similar RT-PCR results between the two parents. There were 6 genes that showed significant differential expression after Al^3+^ exposure between the two parents ZH 24 and HX 3 (Fig 6). *Glyma*.*04g218700*, which encodes a WRKY transcription factor, was dramatically induced by aluminum stress but exhibited decreased expression in the absence of Al^3+^ treatment. *Glyma*.*04g212800*, *Glyma*.*04g213300* and *Glyma*.*04g217400* were markedly upregulated in ZH 24, with more than a 5-10-fold increase in gene expression. Likewise, *Glyma*.*04g216100* and *Glyma*.*04g220600* showed higher expression levels in HX 3 under conditions of Al^3+^ treatment than that in the control treatment (Fig 6). Therefore, our results suggested that these candidate genes may play important roles in the response to aluminum stress in soybean.

## Discussion

### RRE correlates with AAC content in soybean

Evaluation of the phenotypic characteristics of aluminum tolerance of soybean is challenging due to the complex variations among interacting factors and multiple tolerance mechanisms. Different screening methods for phenotypic identification have been proposed to elucidate the inheritance of Al^3+^ tolerance in diverse genotypes [47–50]. Hydroponic cultivation is preferred as feasible method for estimation of Al^3+^ resistance that achieves consistent modulation of conditions, while sand cultivation is thought to mimic the actual growth environment [51, 52]. In addition, researchers commonly use physiological and morphological indicators to indirectly determine Al^3+^ tolerance in spite of disparate growth stages [5, 48, 53]. RRE has been considered to be the most reliable indicator of Al^3+^ tolerance under solution culture conditions in crop seedlings and has been successfully used for genetic analysis of RIL populations in rice [54], maize [20], wheat [55, 56], and soybean [24]. Kopittke *et al*. demonstrated that root elongation could be inhibited by only 30 min of exposure to 30 μM [Al^3+^] and experienced 76% inhibition after 48 h of treatment. In our study, the average inhibition rate for root elongation in our lines was 29% when treated with 25 μΜ [Al^3+^] for 24 h, indicating a reasonable hypothesis consistent with that of Kopittke [57].

On the other hand, root regeneration length (RRL), hematoxylin staining and root dry weight have been widely used as indicators for the assessment of Al^3+^-tolerant cultivars [58–62]. Hematoxylin staining technique was reported to be an efficient method for determination of Al^3+^ tolerance in barley [59], which indicates a specific association between AAC and Al^3+^ tolerance. But AAC has not been widely used to evaluate Al^3+^ tolerance in soybean RIL populations. In our study, AAC was applied as an indicator for QTL mapping of Al^3+^ tolerance in RILs population of soybean. A significant negative correlation was observed between RRE and AAC in a large population of 160 RILs (Fig 2). Indeed, the relationship between AAC and Al^3+^-tolerance of plant was closely associated with exclusion mechanisms and internal detoxification mechanisms [5]. Previous studies have shown that Al^3+^-sensitive genotypes accumulated more total Al^3+^ in root apices than Al^3+^-tolerant genotypes [63], and similar results have also been obtained in rice and Arabidopsis [49, 64]. Undoubtedly, exclusion mechanisms support that the plant of higher tolerance always with the less AAC. However, the relationship of internal detoxification and AAC was ambiguous as Al^3+^ might accumulate in vacuole, though which was just one part of detoxification mechanisms [65]. The significant relationship between RRE and AAC in this study indicated a crucial role of the exclusion mechanism in Al^3+^ tolerance. And the correlation degree between RRE and AAC was different among the various lines (Fig 2), which may depend on genotypic differences and the complex coordination of Al^3+^ tolerance mechanisms in soybean.

Moreover, the estimated heritability of RRE was 92.59% indicating the trait tolerant to Al stress using RRE index had high selection efficiency. The heritability of AAC was 64.90% lower than that of RRE, which might be related to the effect of genotype-environment interactions.

### QTLs associated with Al^3+^ tolerance

A practical way to study the genetics of quantitative traits is to construct a genetic linkage map and map QTLs with segregation of populations based on those traits. Several previous studies have detected QTLs for Al^3+^ tolerance using different mapping populations. The two populations named Young × PI 416937 and Kefeng No. 1 × Nannong 1138–2 are favored by researchers. Various QTLs associated with Al^3+^ resistance were identified by improving the genetic linkage map established by RFLP and SSR markers using the same RIL populations [21, 23, 24, 34, 66]. In our study, the stable RIL population exceeded F_12_ generations with diversified segregation, and there were considerable differences between the two parents in terms of Al^3+^ tolerance traits. Moreover, high-density genetic maps have been constructed using the RAD-seq technology with SNP markers and have been applied to multiple traits [35, 36]. These advantages provided a suitable precondition for our research.

In the present study, a genetic map was used to map the QTLs for Al^3+^ resistance in soybean. As a result, a total of five Al^3+^ tolerance QTLs explaining 39.65% of the total variation were mapped on four chromosomes with narrow intervals (Table 2). One of the five QTLs (*qRRE_17*) is close to the SSR marker Satt186 in the genome of Williams 82 version 1.01, which was associated with Al^3+^ tolerance in previous studies [66] (Table 3). The other four QTLs (*qRRE_04*, *qRRE_16*, *qAAC_04*, *qAAC_19*) are novel loci. Bianchihall *et al*. [23] and Abdel-Haleem *et al*. [34] also identified QTLs for Al^3+^ tolerance on Chr. Gm16 and Gm19, which could not be detected here (Table 3). These discrepancies could be ascribed to the different genetic backgrounds and differences in screening methods.

**Table 3:** QTLs detected by previous studies and the present study associated with Al^3+^ tolerance in soybean.

Chr	Interval (a1.v1)	CI (cM)	Physical positions	R^2^(%)	Indicators	Ref.
Gm08	BARC-014837-01682-Satt333	117.50–119.50	35598937–39910959		Tap root extension	Bianchihall *et al*.2000
Gm11	Sat_270-Sat_272	19.00–21.00	4234139–2718892			Bianchihall *et al*.2000
Gm11	Satt638-BARC-042837-08435	39.10–41.10	6971135–8150135			Bianchihall *et al*.2000
Gm13	BARC-045205-08910-SOYHSP176	66.20–68.20	26196486–29041580			Bianchihall *et al*.2000
Gm16	Sat_366-BARC-024047-04716	56.20–58.20	30404629–31474289			Bianchihall *et al*.2000
Gm19	Satt723- BARC-039375-07304	3.10–5.10	264193–843081			Bianchihall *et al*.2000
Gm02	Satt703-LE45	87.50–98.10		24.60	Plant hight No leaves Shoot dry wight Root dry weight	Qi *et al*.2008
Gm02	A516-A953	101.70–109.30		1.90		Qi *et al*.2008
Gm11	GMKF046-GMKF080	65.10–80.60		8.90		Qi *et al*.2008
Gm17	GMKF058-Satt397	108.00–124.00		9.07		Qi *et al*.2008
Gm17	Satt397-satt669	120.50–127.80		6.83		Qi *et al*.2008
Gm19	satt278-sat_195	49.60–57.00		6.01		Qi *et al*.2008
Gm19	satt278-sat_195	42.50–63.70		5.70		Qi *et al*.2008
Gm20	B39-Sat_419	97.10–107.90		10.50		Qi *et al*.2008
Gm06	Satt202-Satt371	126.23–145.47	48441504–49759893	34.00	Root tolerance index Root relative mean growth	Sharma *et al*.2010
Gm13	Satt252-Satt160	16.08–33.18	16454986–17875691	31.00		Sharma *et al*.2010
Gm02	Satt698-BARC-030679-06925	38.04–42.04	8827384–10906849	5.06	Plant dry weight Shoot dry wight Root dry weight	Korir *et al*.2011
Gm09	BARC-042823-08429-BARC-044609-08738	60.32–62.32	19422282–41745478	4.91		Korir *et al*.2011
Gm10	BE801128- Sat_242	68.97–74.05	38957017–39392879			Korir *et al*.2011
Gm11	Satt197-Sat_128	46.38–53.41	8898878–10011307	9.23		Korir *et al*.2011
Gm17	satt514-Sat_001	86.42–95.55	18425834–36745724	6.64		Korir *et al*.2011
Gm19	Satt313- Satt284	34.54–38.16	34753106–35672961	7.53		Korir *et al*.2011
Gm02	Satt005	75.29	30874668		Relative root elongation	Korir *et al*.2013
Gm06	Satt286	101.75	16171860			Korir *et al*.2013
Gm08	Satt209	128.44	42190891	8.36		Korir *et al*.2013
Gm09	Sct_190	77.37	39455480	6.38		Korir *et al*.2013
Gm10	GMES1703	60.60				Korir *et al*.2013
Gm11	Sat_364	84.25	31594010	8.92		Korir *et al*.2013
Gm13	Sat_240	25.58	1346775	5.73		Korir *et al*.2013
Gm17	Satt186	92.23	39047273–39047329	16.54		Korir *et al*.2013
Gm20	Sat_174	36.59	24547862			Korir *et al*.2013
Gm03	Satt237-K494_1	101.31		10.30	Tap root extension at HIAL Tap root extension at NOAL Relative root extension	Abdel-Haleem *et al*.2014
Gm08	BARCSOYSSR_08_1664-Satt409	152.07		44.80		Abdel-Haleem *et al*.2014
Gm16	B122_1-Satt431	39.01		12.80		Abdel-Haleem *et al*.2014
Gm16	Sat_093-Satt431	38.01		8.80		Abdel-Haleem *et al*.2014
Gm18	Satt570-Satt501	50.03		9.40		Abdel-Haleem *et al*.2014
Gm19	A169_1-A106_1	1.99		5.60		Abdel-Haleem *et al*.2014
Gm04	bin93-bin94	90.50–92.30	45290936–46017212	7.09	Relative root elongation Apical Al^3+^ content	**In this study**
Gm04	bin93-bin94	90.50–92.30	45290936–46017212	8.98		**In this study**
Gm16	bin8	20.30	4038850–4099407	7.80		**In this study**
Gm17	bin93	89.70	38592282–38624563	8.52		**In this study**
Gm19	bin19	27.10	10253846–10556775	0.07		**In this study**

Notably, *qRRE_04* or *qAAC_04* are located in the same bin marker on Chr. Gm04, indicating that it could be an important locus for Al^3+^ resistance (Fig 5). Moreover, *qRRE_04* is a colocalized QTL that overlaps with the QTL for low-P stress from Zhang et al. [67]. Similar results were also obtained by QTL mapping for Al^3+^ tolerance in common bean [68]. Recent research has shown that phosphorus application could reduce aluminum toxicity [69]. Taken together, these results demonstrated that there are some QTLs in the colocalized intervals that may be associated with Al^3+^ tolerance and P stress in soybean.

### Analysis of candidate genes

A total of 66 genes were predicted in the regions of four mapped QTLs, while 54 genes were predicted in the colocalized loci of *qRRE_04* (S5 Table). Six genes showed more significant differential expression under Al^3+^ treatment in both ZH 24 and HX 3 than other candidate genes (Fig 6). None of the genes have been studied in terms of Al^3+^ tolerance, but some were mentioned in previous reports. *Glyma*.*04g218700*, a member of the WRKY transcription factors, named *WRKY21* by Zhou *et al*. [42], was reported to respond to cold stress. In the present study, *Glyma*.*04g218700* was strongly induced after Al^3+^ exposure, especially in HX 3 (Fig 6). *Glyma*.*04g217400*, encoding the ethylene-responsive transcription factor ABR1, may be closely associated with abiotic stress because the homologous gene *AtABR1* can be induced by chilling, salt stress and drought stress in Arabidopsis, showing a strong response to ABA (Fig 6) [70]. *Glyma*.*04g213300* (NAC) and *Glyma*.*04g216100* (Trihelix), encoding two transcription factors, were also reported to respond to multiple forms of abiotic stress [71, 72]. In addition, an upregulated gene, *Glyma*.*04g220600*, was recorded to encode a peroxidase (POD) activated by reactive oxygen species (ROS), which may contribute to Al^3+^ resistance by inducing the production of ROS in plant roots [73]. We also observed that the *Glyma*.*04g212800* gene upregulated by aluminum stress encodes a GDP-mannose transporter with an ambiguous relationship between GDP-mannose transport and aluminum stress. Thus, these 6 genes were identified as candidate genes for aluminum stress tolerance based on the potential response to other forms of abiotic stress (Fig 6). Among the six genes, *Glyma*.*04g218700* may be the strongest candidate gene for aluminum stress tolerance.

## Conclusions

In summary, an RIL population derived from ZH 24 × HX 3 was used to investigate the quantitative inheritance of RRE and AAC for Al^3+^ tolerance in soybean. A high-density soybean genetic map was constructed using 2,639 recombination bin markers by the RAD-seq approach to identify QTLs. A total of five QTLs (*qAAC_04*, *qRRE_04*, *qRRE_16*, *qRRE_17* and *qAAC_19*) were mapped on four chromosomes (Chr. Gm04, Gm16, Gm17, and Gm19) with comprehensive genetic effects of 39.65%. The QTLs *qRRE_04* and *qAAC_04* could be detected by the same markers in a genetic region between 90.50 and 92.30 cM, indicating a new QTL on Chr.04 for the Al^3+^ tolerance trait of soybean. In addition, 66 annotated genes were predicted in the regions of the five QTLs, with six genes showing significantly different expression after Al^3+^ exposure by qRT-PCR. *Glyma*.*04g218700*upregulated by Al^3+^ treatment with the highest expression level may be a candidate gene with potential roles in the response to aluminum stress. Therefore, our efforts will enable future functional analysis of candidate genes and will contribute to the strategies for improvement of aluminum tolerance in soybean.

## Supporting information

S1 Fig Distribution of SNP loci on 20 chromosomes based on the ZH 24 × HX 3 RIL population. (TIF)

S2 Fig Schematic and distribution of bin markers on 20 chromosomes based on the ZH 24 × HX 3 RIL population. (TIF)

S3 Fig Expression analysis by RT-PCR for differentially expressed genes. RT-PCR analysis was carried out using the two parents ZH 24 or HX 3 under the conditions with or without Al^3+^ treatment. There were 15 differentially expressed genes between the conditions with and without Al^3+^ treatment, with the soybean gene *β-Tubulin* as the internal reference.
(TIF)

S1 File Twenty linkage groups of the soybean high-density genetic map of the ZH 24 × HX 3 RIL population and the positions of QTLs for two traits. (PDF)

S1 Table Primers for the RT-PCR of genes detected in QTL regions. A total of 53 out of 66 predicted genes could be amplified successfully, and the other 13 predicted genes could not be amplified, had very short sequences or were not true genes.
(XLSX)

S2 Table Primers for qRT-PCR of differentially expressed genes identified by RT-PCR. (XLSX)

S3 Table Phenotypic data of Al^3+^ tolerance traits in two parents and RIL lines. (XLSX)

S4 Table Segregation distortion of 2639 bin markers among the RIL population. (XLSX)

S5 Table Annotation analysis of all 66 genes detected in QTL regions. (XLSX)

S6 Table Gene ontology (GO) enrichment analysis of genes in QTLs. (XLSX)

## Abbreviations

RIL: Recombinant inbred line
QTL: Quantitative trait locus
CIM: Composite interval mapping
RRE: Relative root elongation
AAC: Apical Al^3+^ content
Chr: Chromosome
LOD: Log-likelihood
RFLP: Restriction fragment length polymorphism
AFLP: Amplified fragment length polymorphism
SSR: Simple sequence repeats
SNP: Single-nucleotide polymorphism
RAD-seq: Restriction-site-associated DNA sequencing
NGS: Next-generation sequencing
