# Supplementary figures and images for: QTL mapping for aluminum tolerance in RIL population of soybean (Glycine max L.) by RAD sequencing
Source: PLoS One. 2019 Oct 29;14(10):e0223674. doi: 10.1371/journal.pone.0223674 (PMC6818782; doi:10.1371/journal.pone.0223674)

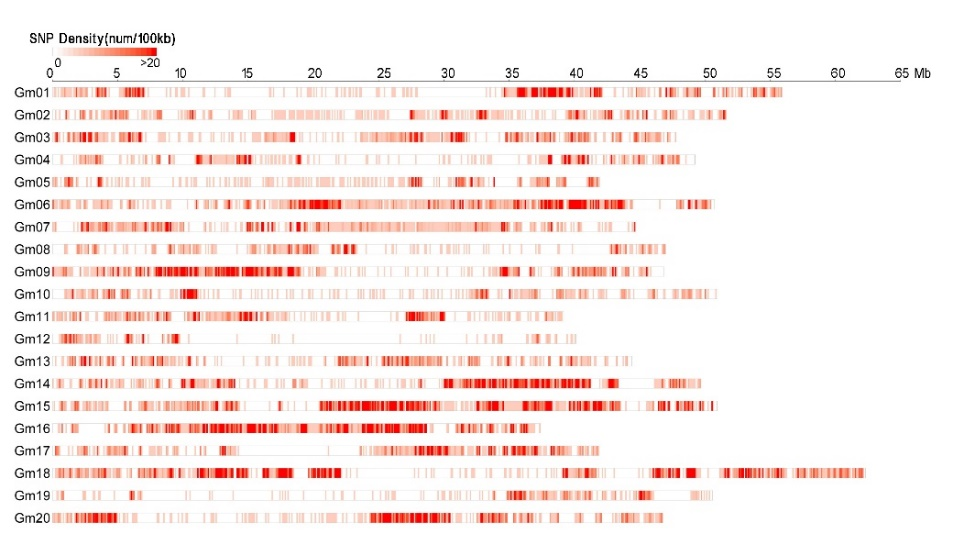

Supplement: S1 Fig — (TIF) [file pone.0223674.s001.tif]

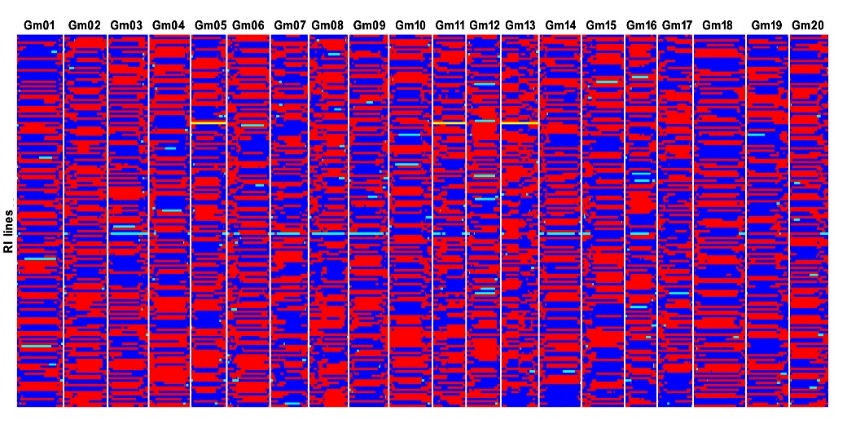

Supplement: S2 Fig — (TIF) [file pone.0223674.s002.tif]

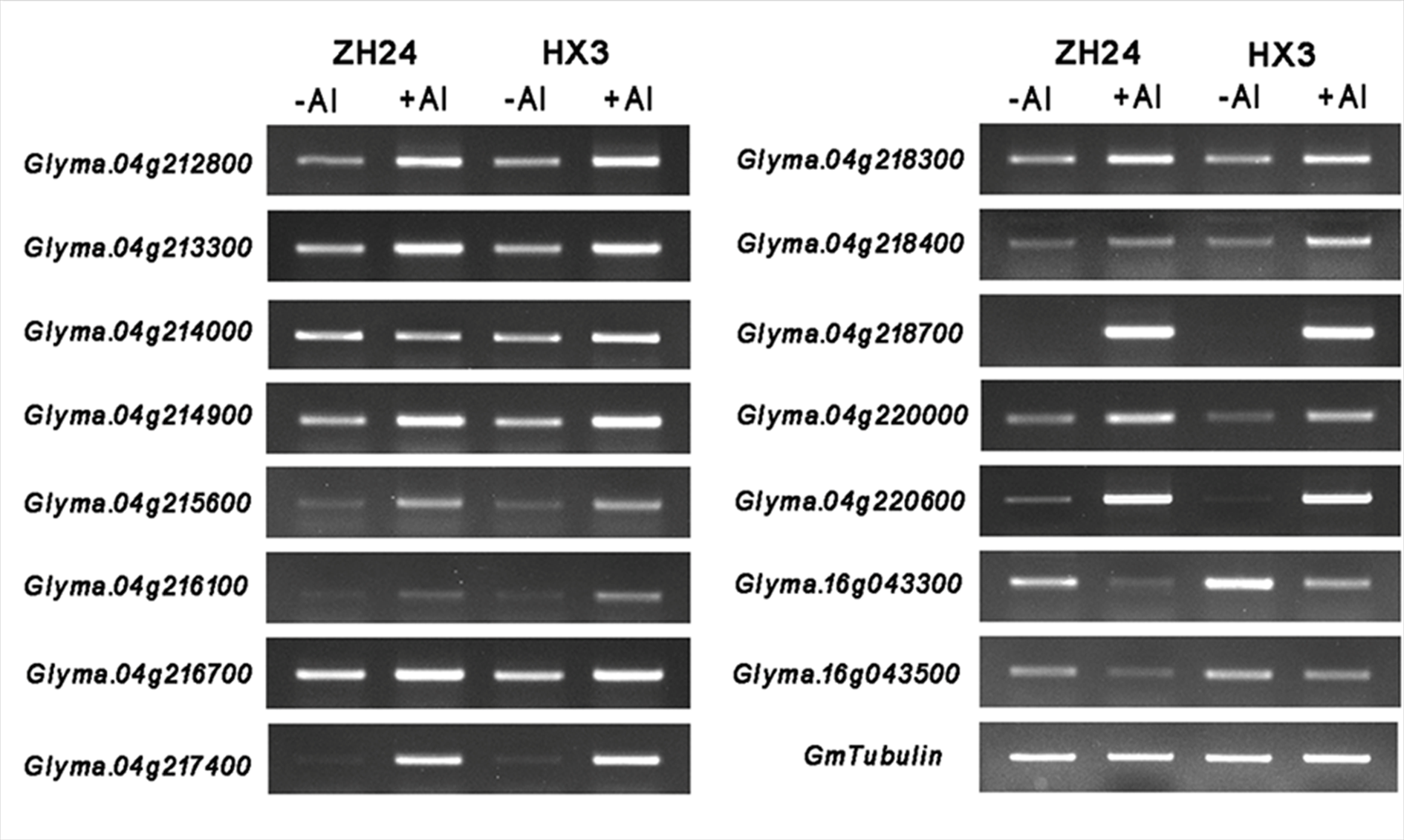

Supplement: S3 Fig — RT-PCR analysis was carried out using the two parents ZH 24 or HX 3 under the conditions with or without Al3+ treatment. There were 15 differentially expressed genes between the conditions with and without Al3+ treatment, with the soybean gene β-Tubulin as the internal reference. (TIF) [file pone.0223674.s003.tif]

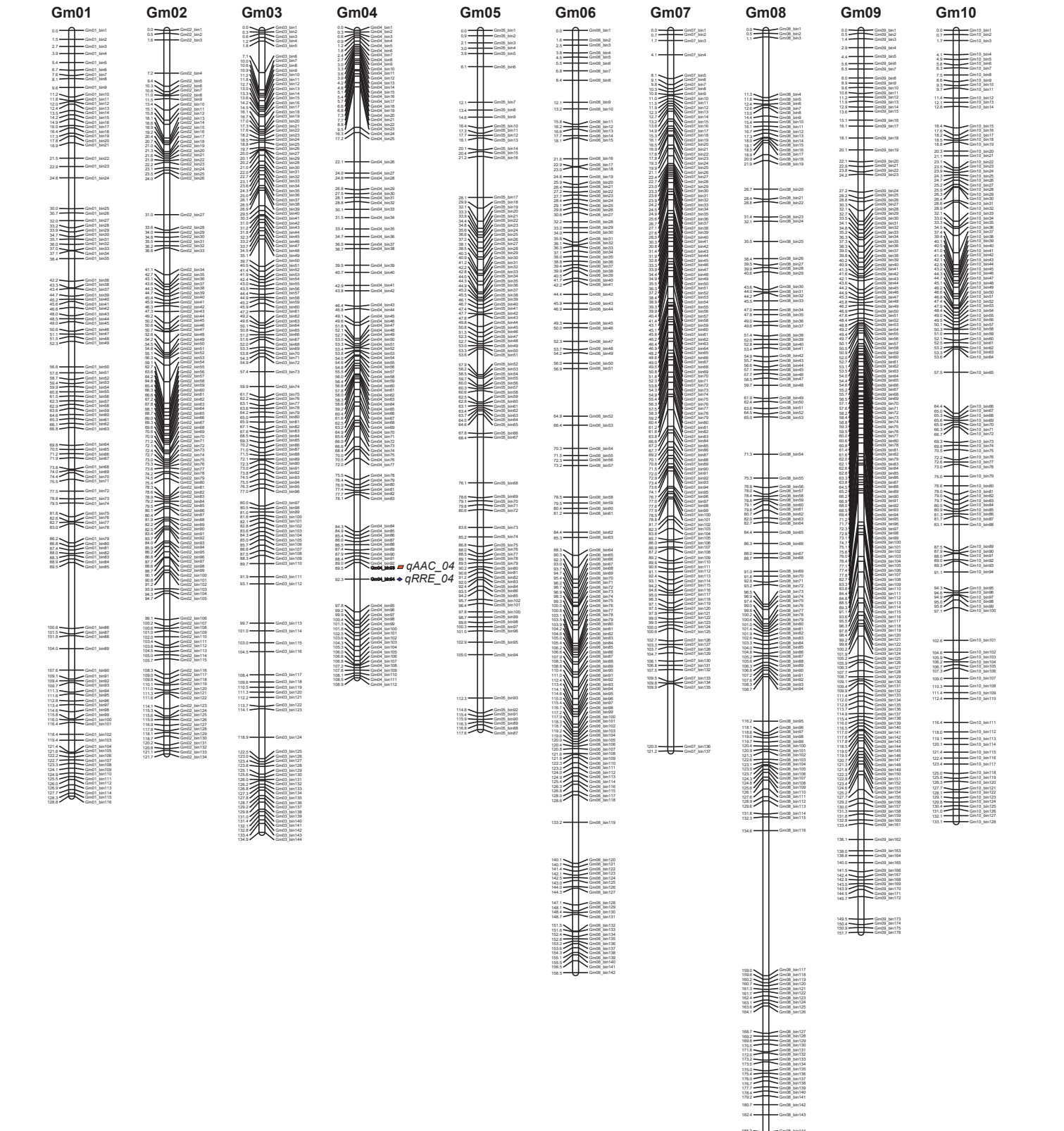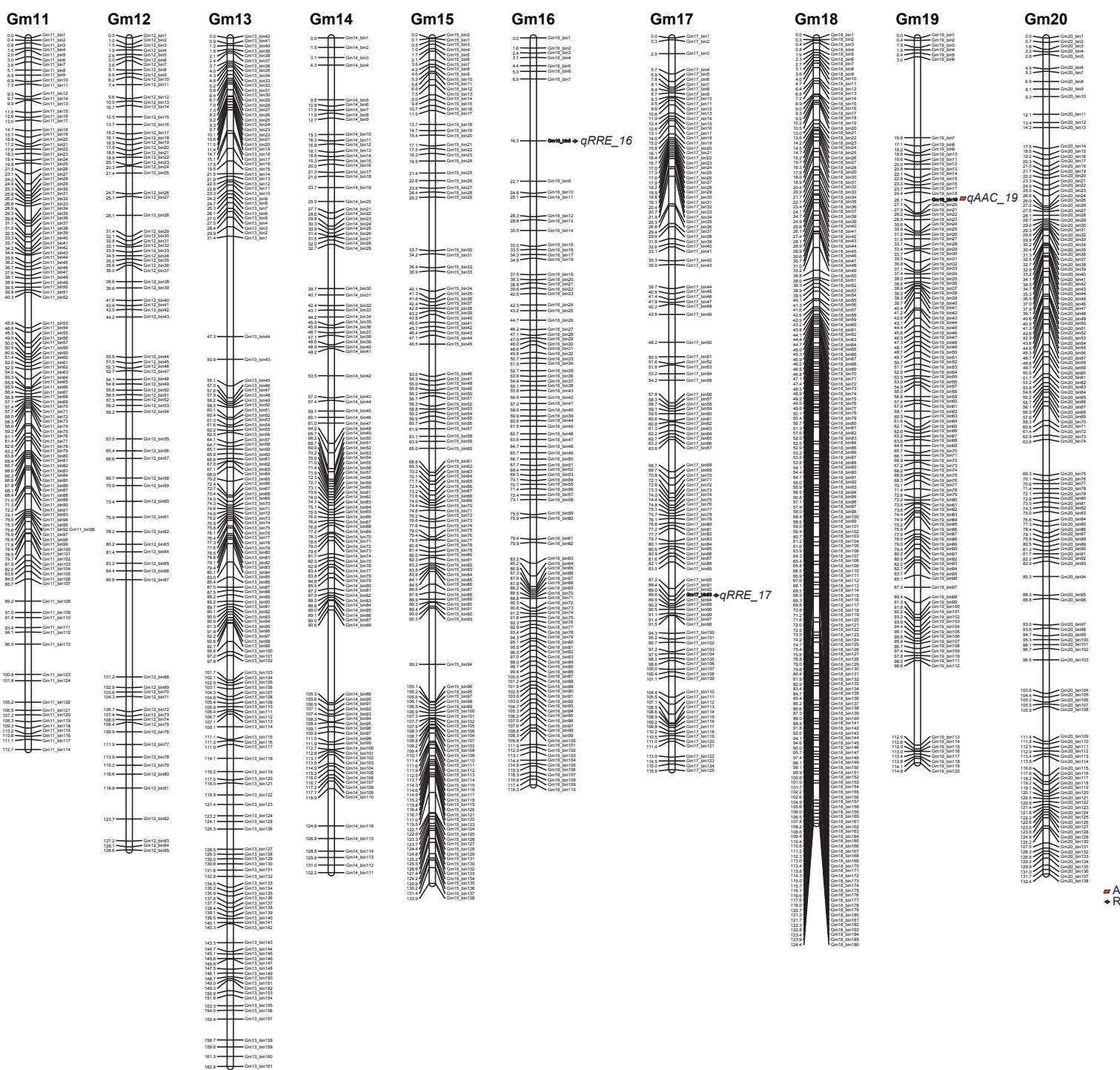

Supplement: S1 File — (PDF) [file pone.0223674.s004.pdf]
